# Caspase-3 cleaves and activates the NADase SARM1 to promote apoptosis, linking two cell death mechanisms

**DOI:** 10.1073/pnas.2528118123

**Published:** 2026-01-23

**Authors:** Jianjin Shi, Ye Eun Kim, Nicolás José DeRuiter, Priyanka Kadav, Marc Tessier-Lavigne

**Affiliations:** ^a^Department of Biology, Stanford University, Stanford, CA 94305

**Keywords:** SARM1, apoptosis, axon degeneration, caspase

## Abstract

Two major mechanisms of axon degeneration have been identified. The first, a caspase-dependent apoptotic pathway, is a major mediator of developmental axon degeneration triggered by loss of trophic support. The second, a caspase-independent pathway mediated by the sterile alpha and HEAT/Armadillo motif containing 1 (SARM1) NADase, was found in studies of injury-induced Wallerian degeneration; it is also implicated in degeneration associated with traumatic brain injury, as well as some neurodegenerative diseases and neuropathies. Recent studies suggest that SARM1 functions as a metabolic sensor for the cellular nicotinamide mononucleotide/NAD+ ratio through its autoinhibitory armadillo repeats (ARM) domain. Here, we show a tight link between apoptotic and SARM1-dependent degeneration by demonstrating that SARM1 is activated during and contributes to apoptosis in neuroblastoma cells, macrophages, and T cells. Mechanistically, the key apoptotic protease caspase-3 cleaves SARM1 within its ARM domain, relieving its autoinhibition and activating its NAD+ hydrolase activity. Using a knock-in (KI) mouse model with a SARM1 mutation that prevents caspase-3 cleavage, we show that apoptosis promotion by SARM1 in macrophages and T cells requires its cleavage, whereas in neurons deprived of trophic support, activation of SARM1 occurs both with and without cleavage. Our study identifies a central role for SARM1 in apoptosis in some cells that is mediated by SARM1 activation through caspase-3 cleavage; it provides a model for dissecting the contributions of the two modes of SARM1 activation in different cellular contexts; and it has implications for the selection of ortho- versus allosteric SARM1 inhibitors for treating neurodegenerative diseases.

## Significance

Sterile alpha and HEAT/Armadillo motif containing 1 (SARM1), an NAD+ hydrolase, drives axon degeneration/cell death in injured axons and in many neurodegenerative disease models. SARM1 can be activated by sensing a rise in the nicotinamide mononucleotide (NMN)/NAD+ ratio in cells/axons but other activation mechanisms remain understudied. This study identifies a critical role of SARM1 in promoting apoptosis. During apoptosis, the key apoptotic protease caspase-3 cleaves SARM1 within its autoinhibitory armadillo repeats (ARM) domain, derepressing its intramolecular autoinhibition and activating its NAD+ hydrolase function. This study proposes a mechanism of SARM1 activation by caspase-3 cleavage and provides a knock-in mouse model that will help dissect the contribution of this mechanism of SARM1 activation in disease models.

The nervous system relies on a complex network of axonal connections among neurons. Under stress or following injury, axons can undergo degeneration, an active self-destructive process (1). Two main mechanisms of axon degeneration have been extensively studied: a conventional caspase-dependent apoptotic pathway and a caspase-independent, injury-induced pathway known as Wallerian degeneration (2). Axon degeneration is implicated in various conditions, including traumatic injuries, neurodegenerative diseases, and peripheral neuropathy (3).

SARM1 has traditionally been associated with promotion of injury-induced axon degeneration (4, 5). Genetic deletion of SARM1 in models such as flies or mice provides lasting protection to injured axons, both in vitro and in vivo (4). SARM1’s Toll, Interleukin 1 receptor, and Resistance protein (TIR) domain possesses NAD+ (nicotinamide adenine dinucleotide, oxidized form) hydrolyzing enzymatic activity (6). When SARM1 is directly activated, it depletes NAD+ and ATP, triggering cell death and axon degeneration (7, 8). Targeting SARM1 or its pathway has shown therapeutic promise in models of traumatic brain injury, chemotherapy-induced peripheral neuropathy (CIPN), and other conditions (9–17).

SARM1 also promotes T cell death after immune activation (18) and neuron cell body death in response to viral infection (19, 20), indicating that, in addition to its well-studied role in axon degeneration, SARM1 participates in cell death of both neuronal and nonneuronal cells.

At a molecular level, SARM1 has been shown to form octameric, ring-shaped oligomers, and is maintained in an autoinhibited state by binding of its armadillo repeats (ARM) domain to its TIR domain to inhibit NADase activity (21–23). How SARM1 is activated physiologically is an area of active study. One mode of activation is proposed to involve detection by SARM1 of increases in the nicotinamide mononucleotide (NMN)/NAD+ ratio through its ARM domain that alters ARM binding to the TIR domain and thereby disinhibits NADase activity (24). Recently, it was reported that a panel of pyrodine-derived SARM1 inhibitors can also activate SARM1 NADase and trigger axon degeneration when provided at subinhibitory concentrations (25–27). The activation mechanism in this case appears to involve formation of adducts of ADP-ribose with these compounds catalyzed by SARM1’s base exchange activity, which trigger formation of superhelical filaments of SARM1 in which the NADase is activated (27). It was proposed that endogenous counterparts to the chemical inhibitors might mediate activation of SARM1 physiologically (27).

To further explore SARM1 activation mechanisms, we set out to develop models for SARM1-dependent degeneration in systems that are more easily tractable biochemically than primary neurons. During these studies, we unexpectedly found that SARM1 contributes significantly to apoptotic cell death in macrophages, T cells, and other cell types. Furthermore, we found that during apoptosis, the key apoptotic effector protease caspase-3 cleaves SARM1 in its ARM domain, derepressing its intramolecular autoinhibition and activating its NAD hydrolase function. We show that cleavage by caspase-3 is essential for SARM1’s role in apoptosis in those cells. In primary neurons, in contrast, while caspase-3 and SARM1 both contribute to degeneration triggered by trophic factor (NGF) deprivation, in this case SARM1 appears to be activated both by caspase-3 and in parallel by a caspase-independent pathway.

Our findings identify an important role for SARM1 downstream of caspase-3 in apoptotic cell death in some cells and reveal a previously unsuspected mode of SARM1 activation by cleavage. They also show that in other cells, caspase-3 and SARM1 can collaborate in parallel to trigger degeneration.

## Results

### SARM1 Promotes Apoptosis in a Neuronal Cell Line and in Primary Cells.

To facilitate biochemical analysis, we explored how SARM1 functions in cells other than primary neurons. We examined a panel of established cell lines to identify any that express *Sarm1*, which we presumed to be a prerequisite to SARM1 sensitivity.

**Fig. 1.**
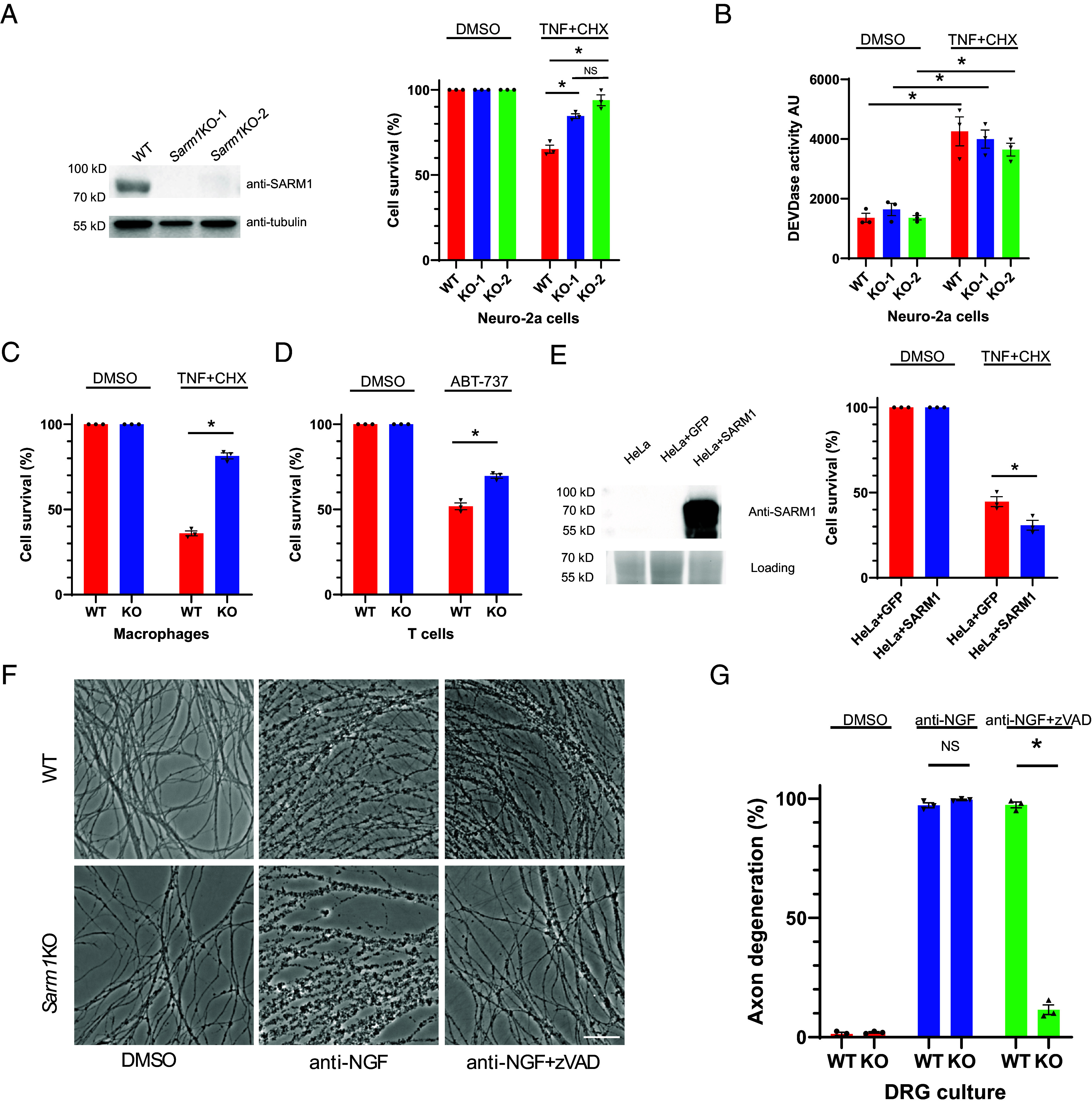
SARM1 promotes apoptotic cell death. (*A*) Neuro-2a cells show defective apoptosis. WT Neuro-2a cells and two single-cell derived *Sarm1* KO lines (KO-1 and KO-2, generated by CRISPR-Cas9 mediated targeting) were treated either with DMSO or with TNF-α (20 ng/mL, TNF) combined with cycloheximide (10 µg/mL, CHX) for 16 h. Percent cell survival was determined by measuring total ATP level and normalizing to control (DMSO) treatment. Data are from three biological replicates. The two KO lines were verified by Western blotting of SARM1 (*Left*) using a monoclonal antibody targeting the amino terminus of SARM1 (clone 10G2, *SI Appendix*, Fig. S4). Tubulin Western blot was used as loading control. Western blot data shown are representative of three independent experiments. (*B*) Caspase-3/7 activity (i.e., DEVDase activity) following TNF and CHX cotreatment was measured using the caspase-glo kit from Promega. AU, arbitrary units. Data are from three biological replicates. (*C* and *D*) effect of SARM1 on apoptosis of macrophages and T cells. (*C*) primary bone marrow derived macrophages were treated with DMSO or TNF (10 ng/mL) and CHX (10 µg/mL) for 7 h. (*D*) primary T cells were treated with ABT-737 (1 µM) for 23 h. Percent cell survival was determined by measuring total ATP level and normalized to control (DMSO) treatment. Data are from three biological replicates. (*E*) SARM1 expression sensitizes HeLa cells to apoptosis. HeLa cells stably expressing GFP (HeLa+GFP) and HeLa cells stably expressing SARM1 (HeLa+SARM1) were treated with TNF+CHX for 18 h. Percent cell survival was determined by measuring total ATP level and normalized to control (DMSO) treatment. Data are from three biological replicates. SARM1 expression was confirmed by Western blot (*Left*). A portion of the stain free gel image was used as an internal loading control. Western blot data shown are representative of three independent experiments. (*F* and *G*) *Sarm1* KO blocks anti-NGF-induced axon degeneration in the presence of caspase inhibitor. (*F*) Representative images of WT and *Sarm1* KO DRG cultures (7 d in vitro, DIV) treated with DMSO, anti-NGF, or anti-NGF together with zVAD-FMK (100 µM, zVAD) for 22 h. (Scale bar, 50 µm.) Images are representative of three independent experiments. (*G*) Axon degeneration percentage of (*F*) was quantified and shown in the bar graphs. Data are from three biological replicates. In all panels, Student’s *t* test was used to determine statistical significance. * Indicates *P* < 0.05, NS, nonsignificant. Error bars represent SEM.

We found that Neuro-2a, a neuroblastoma cell line, exhibits high endogenous *Sarm1* expression. To assess a role for SARM1 in cell death, we generated *Sarm1* knock-out (KO) Neuro-2a cells using CRISPR-Cas9 (Fig. 1*A*), enabling us to assess directly whether potential cell death inducers are SARM1-dependent by comparing responses of the KO and parental lines.

We next needed to identify a way of activating SARM1 in these cells. We first explored whether induction of mitochondrial dysfunction, a known activator of SARM1 in axons (7), can trigger SARM1-dependent cell body death in Neuro-2a cells. We tested two mitochondrial toxins, rotenone and CCCP (carbonyl cyanide m-chlorophenyl hydrazine), both of which induce SARM1-dependent axon degeneration and cell death in primary sensory neurons (7). Both compounds induced robust cell death in wild-type (WT) Neuro-2a cells (*SI Appendix*, Fig. S1). However, the *Sarm1* KO Neuro-2a cells were just as sensitive as the WT cells to these compounds (*SI Appendix*, Fig. S1). These data suggest that, unlike in axons, rotenone and CCCP-induced cell death in Neuro-2a cells does not rely noticeably on SARM1 function.

In a further search for triggers of SARM1-dependent death in Neuro-2a cells, we cast a broader net and screened a panel of other cell death–inducing reagents, including various small molecule compounds and antibodies (see list in *Methods*), comparing their effects on the WT and KO lines. We found that a classic apoptosis-inducing combination, tumor necrosis factor alpha (TNF) combined with the protein synthesis inhibitor cycloheximide (CHX), had markedly different effects on WT and *Sarm1* KO Neuro-2a cells (Fig. 1*A*), implicating SARM1’s role in apoptotic cell death in these cells. The combination of TNF and CHX is known to activate extrinsic apoptotic pathways mediated by caspase-8 and caspase-3, and, consistent with this, we observed strong caspase-3 protease (DEVDase) activity in Neuro-2a cells treated with TNF and CHX (Fig. 1*B*). Interestingly, caspase-3 activity was elevated to a similar extent in WT and *Sarm1* KO cells (Fig. 1*B*), indicating that SARM1 functions downstream or in parallel to caspase-3 activation in Neuro-2a cells.

The finding of a role for SARM1 in apoptosis of Neuro-2a cells prompted us to study apoptosis of several other cell types. For this, we used primary cells derived from *Sarm1* KO mice (28).

Previous studies showed that SARM1 is expressed in bone marrow–derived macrophages (29) and primary T cells (18). We found that macrophages from *Sarm1* KO mice displayed resistance to apoptotic cell death caused by TNF and CHX treatment, when compared to WT cells (Fig. 1*C*). We also tested TNF and CHX on T cells, but, in contrast to macrophages, this treatment triggered only a modest degree of apoptosis in those cells. We therefore turned to another apoptotic inducer compound, ABT-737, which functions to disinhibit Bax and activates a caspase cascade (30). ABT-737 triggered a significant degree of apoptosis in T cell (Fig. 1*D*). As in macrophages, T cells from *Sarm1* KO mice displayed resistance to this cell death (Fig. 1*D*), implicating SARM1 functions in apoptosis in these cells as well.

We next performed a gain-of-function study to ask whether addition of SARM1 to nonexpressing cells could augment an apoptotic response. We found that human cervical cancer-derived HeLa cells have no detectable SARM1 expression natively. However, after stably expressing SARM1, these cells showed a significant increase of apoptotic cell death compared with control HeLa cells when challenged with apoptotic triggers (Fig. 1*E*).

Collectively, these findings establish a critical role for SARM1 in promoting apoptosis in Neuro-2a cells, primary macrophages and T cells, and that SARM1 can augment apoptosis when added exogenously to HeLa cells.

NGF-deprivation is known to trigger a traditional caspase-dependent apoptotic pathway in cultured sensory neurons and their axons (30). Different studies have implicated SARM1 to different extents in this form of axonal degeneration, likely due to differences in precise neuronal culture conditions (*Discussion*). To test whether SARM1 contributes to axon degeneration in our sensory neuron culture paradigm, we treated WT and *Sarm1* KO dorsal root ganglion (DRG) sensory neuron cultures with anti-NGF antibody to deplete NGF, resulting in swift degeneration of their axons and death of their cell bodies. WT and *Sarm1* KO axons appeared to degenerate equally rapidly in these cultures (Fig. 1 *F* and *G*). In addition, under these culture conditions, the pan-caspase inhibitor zVAD-FMK by itself did not have a noticeable effect on degeneration of WT axons (Fig. 1 *F* and *G*). However, the combination of zVAD-FMK and knocking out *Sarm1* resulted in strong axon protection (Fig. 1 *F* and *G*). Thus, in our assay, SARM1 and caspases collaborate to mediate degeneration, providing another system to probe the interplay of apoptosis and SARM1-mediated cell death.

### Caspase-3 Cleaves SARM1 In Vitro.

**Fig. 2.**
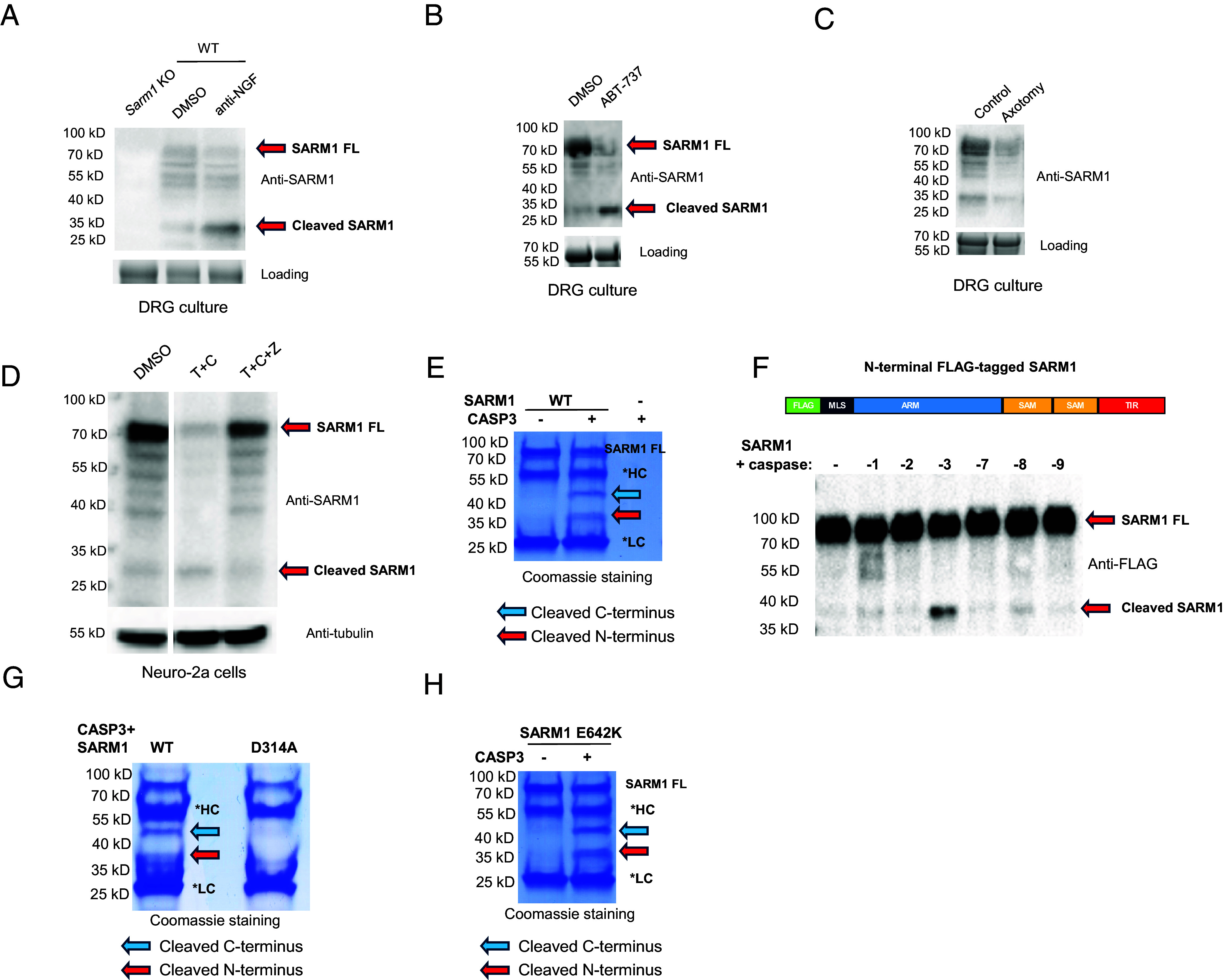
SARM1 is cleaved by active caspase-3. (*A*) DRG cultures were treated with anti-NGF for 16 h. Western blot of SARM1 protein shows reduction of full-length SARM1 (SARM1 FL) and increase of a band of ~33 kDa. A *Sarm1* KO sample was used to demonstrate the specificity of the SARM1 antibody (*Left* lane). A portion of the stain free gel image was used as an internal loading control. Data are representative of three independent experiments. (*B*) SARM1 from DRG cultures treated with ABT-737 (10 µM, 6 h) also shows a decrease in full-length SARM1 and increase in the ~33 kDa band. A portion of the stain-free gel image was used as an internal loading control. Data are representative of three independent experiments. (*C*) Western blot of SARM1 from control DRG cultures and following axotomy (6 h) shows that SARM1 is not cleaved during Wallerian degeneration. A portion of the stain free gel image was used as an internal loading control. Data are representative of three independent experiments. (*D*) Neuro-2a cells were treated with DMSO (control) or with TNF+CHX for 18 h to induce apoptosis. As in primary neurons, there is a reduction of full-length SARM1 and increase of a band of ~33 kDa. Both effects are blocked by the pan-caspase inhibitor zVAD. T + C: TNF+CHX, T + C + Z: TNF+CHX+zVAD. (Full Western blot shown in *SI Appendix*, Fig. S2). Tubulin Western blot was used as loading control. Data are representative of three independent experiments. (*E*) In vitro cleavage of recombinant SARM1 by active caspase-3. Purified SARM1 (on beads) was incubated with active caspase-3. Cleavage was determined by protein electrophoresis and Coomassie staining. *HC, anti-Flag antibody heavy chain conjugated on beads, *LC: anti-Flag antibody light chain conjugated on beads. Data are representative of three independent experiments. (*F*) Western blot of the caspase-cleaved SARM1 samples using antibody targeting the N-terminal FLAG tag. Data are representative of three independent experiments. Diagram shows the location of the FLAG tag at the N terminus of SARM1. (*G*) Effect of the SARM1 D314A mutation on caspase-3 cleavage. Recombinant WT SARM1 or SARM1 D314A were incubated with active caspase-3 and cleavage was determined as in *E*. Data are representative of three independent experiments. (*H*) Effect of SARM1 catalytic mutant on caspase-3 cleavage. Data are representative of three independent experiments.

In the course of our analysis of primary neurons deprived of NGF, we observed that endogenous SARM1 appears to undergo cleavage during degeneration, as we observed increase of a band of approximately 33 kDa concomitant with decrease in full-length SARM1 (Fig. 2*A*). Since the antibody used to detect SARM1 here recognizes an epitope within its first 27 amino acids (*Methods*), it appears that the ~33 kDa fragment originates from the amino terminus of SARM1. We observed a similar cleavage when apoptosis was activated by exposing the neurons to ABT-737 (Fig. 2*B*). In contrast, no increase in the cleaved band occurred during injury (axotomy)-induced Wallerian degeneration (Fig. 2*C*), consistent with the fact that Wallerian degeneration does not involve caspase activation (30, 31). We also observed a decrease in full-length SARM1 in this condition too, though it was not as great as in the other two conditions and it may reflect the fact that the degenerating axons release their cytosolic contents.

A similar cleavage of SARM1 occurred in Neuro-2a cells triggered to undergo apoptosis by treatment with TNF and CHX. This was inhibited by the pan-caspase inhibitor zVAD-FMK (Fig. 2*D*), indicating that the cleavage is caspase-dependent.

We hypothesized that SARM1 might be directly cleaved by caspases. To test this, we purified recombinant SARM1 protein from HEK293T cells and incubated it with various recombinant caspases. Among all tested caspases, caspase-3, the primary executioner caspase in apoptosis—but not other caspases tested—cleaved SARM1, generating two distinct fragments of approximately 45 kDa and 37 kDa (Fig. 2*E* and *SI Appendix*, Fig. S3). We hypothesized that the smaller 37 kDa fragment includes the N terminus, as its size aligns with the size of the predicted SARM1 N-terminal fragment in combination with an additional 4 kDa due to an N-terminal 3xFLAG tag and linker peptides. This was confirmed when the 37 kDa fragment was recognized by an anti-FLAG antibody (Fig. 2*F*).

**Fig. 3.**
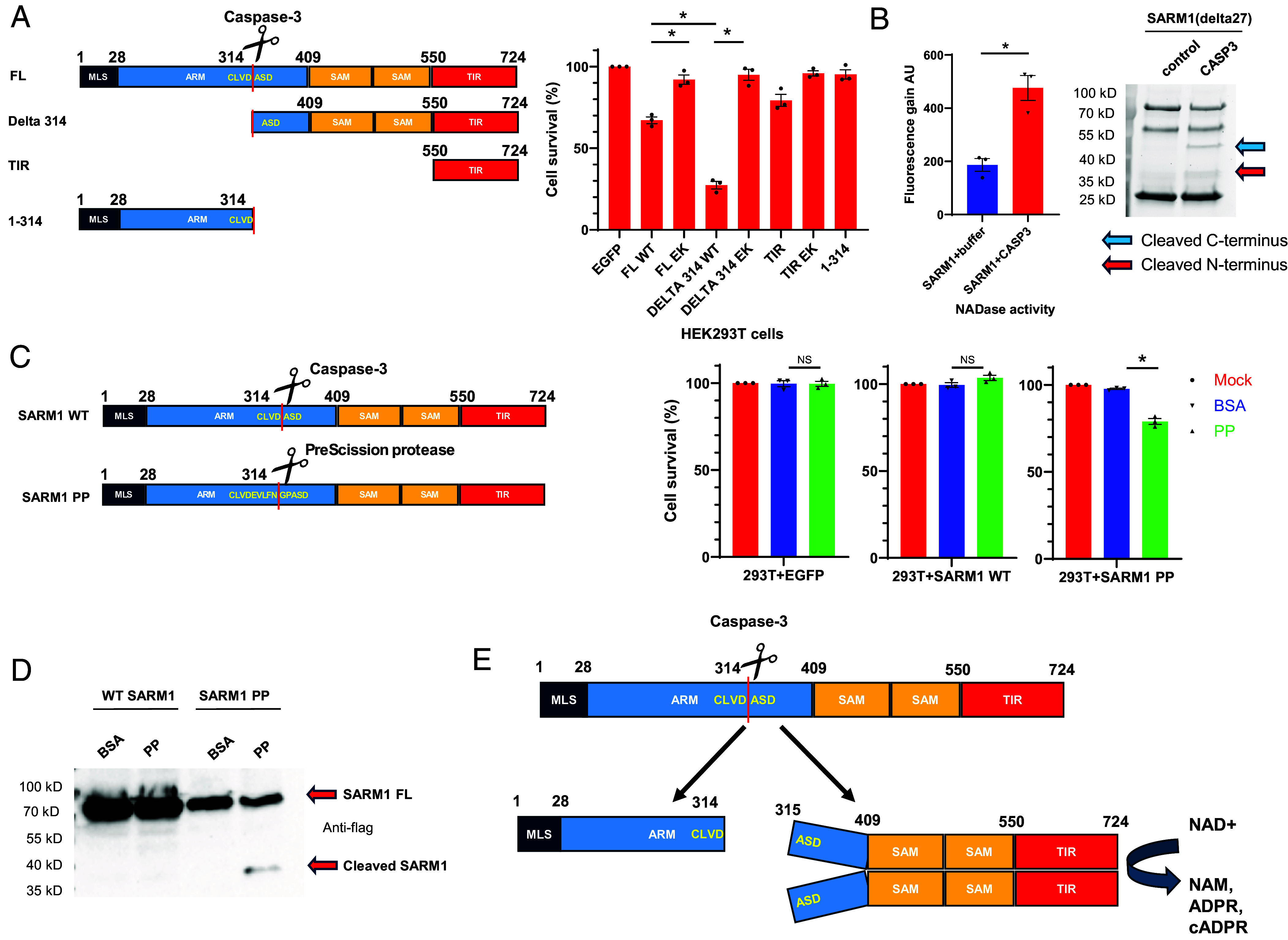
Cleavage activates SARM1. (*A*) Cell death–inducing activity of SARM1 truncations and caspase-3 cleaved fragments. SARM1 fragments, diagrammed on the left, were transiently transfected into HEK293T cells. Cell survival was measured 48 h later using the CellTiter-Glo Luminescent Cell Viability Assay. Percent cell survival was determined by normalization to control (EGFP expressing vector) transfection. Data are from three biological replicates. MLS, mitochondrial localization signal. ARM, armadillo repeats. SAM, sterile alpha motif. TIR, Toll, Interleukin 1 receptor, and Resistance protein domain. (*B*) The effect of SARM1 cleavage on the SARM1 NADase activity. Recombinant SARM1 (lacking its N-terminal 27 amino acids, i.e., SARM1 delta27) was incubated with active caspase-3 (1 unit) or control for 2 h at 37 °C. NADase activity was determined using the ε-NAD assay. AU, arbitrary units. Data are from three biological replicates. The cleavage of SARM1 delta27 was verified using a stain free gel. Stain free gel image was representative of three biological replicates. (*C*) Electroporation of PP triggers cell death of cells expressing engineered SARM1(with PP site, engineered EVLFNGP after D314) but not WT SARM1. EGFP, WT SARM1 or SARM1 PP expressing plasmids were transfected into HEK293T cells for 24 h, then control protein BSA or PP was electroporated into the cells. Percent cell survival was determined 20 h after electroporation by normalizing total ATP level to control electroporation. Data are from three biological replicates. (*D*) Electroporation of PP triggers the cleavage in HEK293T cells of engineered SARM1 containing a PP site (SARM1 PP) but not WT SARM1. SARM1 Western blot image was representative of three biological replicates. (*E*) Diagram of the findings. The caspase-3 cleavage site was determined by Edman degradation of the C-terminal fragment. In all panels, Student’s *t* test was used to determine statistical significance. * Indicates *P* < 0.05, NS, nonsignificant. Error bars represent SEM.

To pinpoint the cleavage site, we performed Edman degradation on the C-terminal 45 kDa fragment, revealing its N-terminal sequence as “_315_ASDTS_319_”, which is located directly after “_311_CLVD_314_”, a conserved sequence in both human and mouse SARM1 (Fig. 3*E*). This matches a potential caspase-3 cleavage site in SARM1 and aligns with the sizes of the resulting fragments. To further confirm that caspase-3 cleaves SARM1 after D314, we created a D314A mutant of SARM1 and found that it was no longer cleaved by caspase-3, as predicted (Fig. 2*G*). An NAD+ hydrolase-inactive SARM1 mutant E642K (6) was still efficiently cleaved by caspase-3, indicating that cleavage is independent of NADase activity (Fig. 2*H*).

### Caspase-3 Cleavage Activates SARM1 NADase Activity.

The finding that caspase-3 cleaves within SARM1’s autoinhibitory ARM domain raised the possibility that this cleavage might release SARM1’s intramolecular inhibition and activate its NADase function.

To test this hypothesis, we expressed each of the two cleavage fragments in HEK293T cells (Fig. 3*A*). The TIR domain alone as well as full-length SARM1 were expressed as controls. As expected, the isolated TIR domain showed minimal cell-killing activity, which previous studies have shown is due to its inability to oligomerize, resulting only in low-level NADase activity. Full-length (FL) SARM1 exhibited moderate cell death–inducing activity, likely due to activation from overexpression. In contrast, the C-terminal cleavage fragment demonstrated robust cell-killing activity, suggesting high-level activation of NADase. Notably, cell-killing activity in all SARM1 truncations was dependent on NADase function, as mutating the catalytic site (E642 to K) completely abolished their effects (Fig. 3*A*).

To further test if the cleavage mediated by caspase-3 directly activates SARM1’s NADase activity, we incubated purified WT SARM1 protein with active recombinant caspase-3 in vitro. Caspase-3 efficiently cleaved SARM1, leading to a marked increase in NADase activity, as shown by biochemical assays (Fig. 3*B*). In this experiment, we used a SARM1 construct lacking the N-terminal 27 amino acids, which contain its mitochondrial localization signal (5, 28), showing that this sequence (and presumably mitochondrial association in vivo) is not required for cleavage.

To determine if cleavage after D314 alone is sufficient to induce SARM1-dependent cell death, we engineered a SARM1 variant with a PreScission protease (PP) recognition sequence (“EVLFNGP”) after D314. To minimize the background level of cell death seen with expression of full-length constructs, in this experiment, we transfected in lower amounts of plasmids. Under these specific conditions, expressing either the engineered SARM1 or WT SARM1 alone did not induce cell death in the time frame provided (Fig. 3*C*). However, when PP was introduced by electroporation, cells expressing the engineered SARM1, but not WT SARM1, underwent significant cell death (Fig. 3*C*), and the engineered SARM1 but not WT SARM1 was cleaved by PreScission protease into fragments of the expected sizes (Fig. 3*D*).

Together, these in vitro and in vivo findings demonstrate that caspase-3 cleavage is sufficient to activate SARM1’s NADase activity.

### A Mouse Model with Uncleavable SARM1 Reveals SARM1’s Contribution to Apoptosis.

**Fig. 4.**
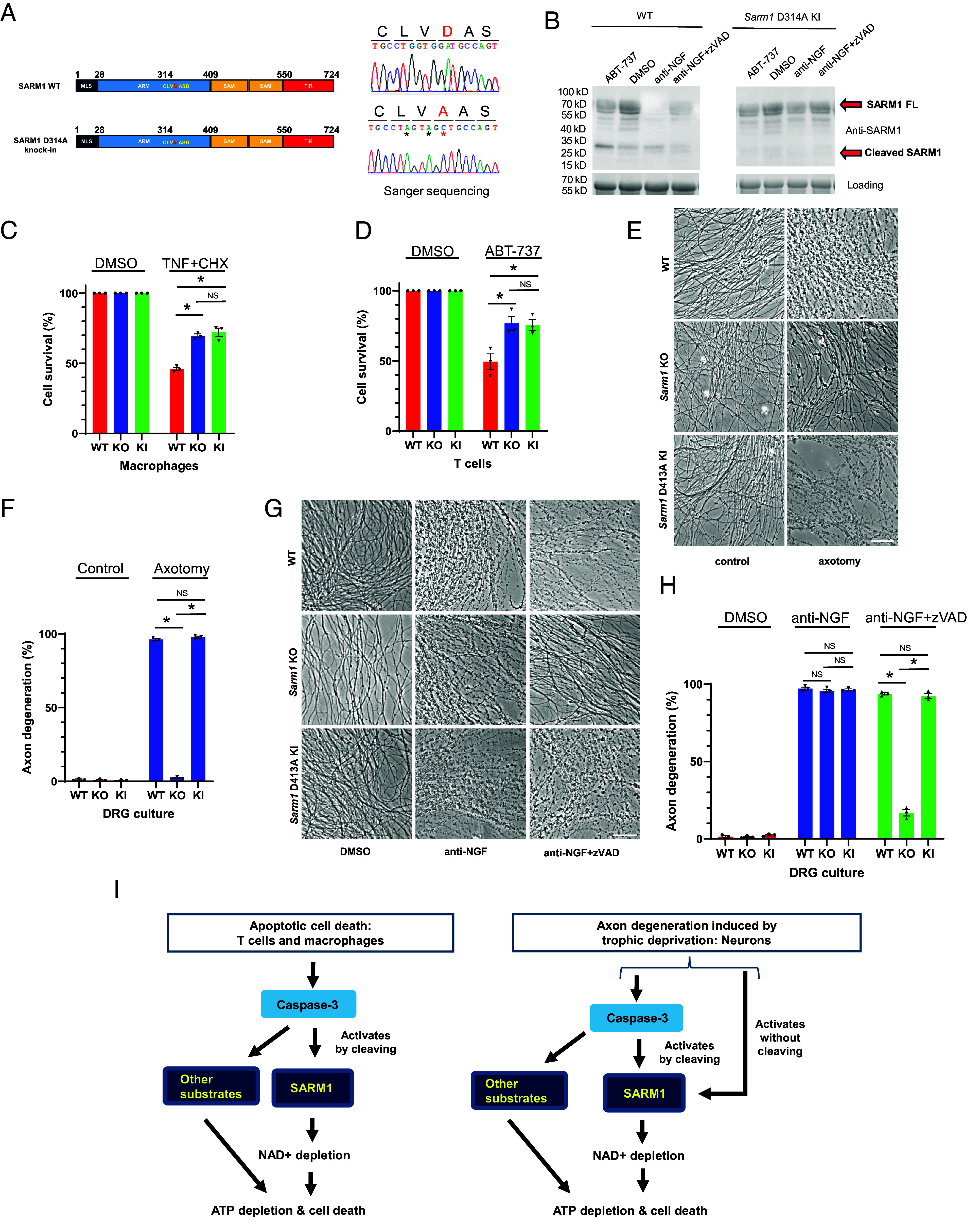
Cleavage is required for SARM1 to promote apoptosis. (*A*) Generation of *Sarm1* D314A KI mice. Diagram shows the intended SARM1 D314A allele. Sanger sequencing of PCR amplified DNA from *Sarm1* D314A KI mice confirmed that D314 was successfully mutated to *A*. Additional same-sense mutations (marked with *) introduced by the KI strategy (*Methods*) were also confirmed. (*B*) SARM1 WT but not D314A is cleaved during apoptosis. Western blot of SARM1 from DRG cultures under different conditions shows that, unlike WT SARM1, SARM1 D314A is not cleaved. Cultures were treated with anti-NGF/anti-NGF+zVAD for 16 h or ABT-737 (10 µM) for 6 h. Data are representative of three independent experiments. (*C*) Effect of SARM1 D314A KI on primary macrophage apoptosis. Cells were treated with DMSO or TNF (10 ng/mL)+CHX (10 µg/mL) for 7 h. Data are from three biological replicates. (*D*) Effect of SARM1 D314A KI on primary T cell apoptosis. Cells were treated with DMSO or ABT-737 (1 µM) for 19 h. Data are from three biological replicates. (*E*) Effect of SARM1 D314A on DRG Wallerian degeneration. Cultures were imaged 6 h after axotomy. (Scale bar, 50 µm.) Images are representative of three independent experiments. (*F*) Axon degeneration percentage of *E* was quantified and shown in the bar graphs. Data are from three biological replicates. (*G*) Effect of SARM1 D314A KI on axon degeneration induced by anti-NGF in the presence of caspase inhibitor. Cultures were imaged 20 h after being treated with DMSO, anti-NGF, or anti-NGF+zVAD. (Scale bar, 50 µm.) Images are representative of three independent experiments. (*H*) Axon degeneration percentage of g was quantified and shown in the bar graphs. Data are from three biological replicates. (*I*) Schematic model illustrating caspase-dependent and -independent activation of SARM1. In all panels, Student’s *t* test was used to determine statistical significance. * Indicates *P* < 0.05, NS, nonsignificant. Error bars represent SEM.

To investigate whether cleavage of SARM1 at D314 is necessary for promoting apoptotic cell death, we generated a *Sarm1* D314A knock-in (KI) mouse line, which was verified by amplification of the target region and sequencing (Fig. 4*A*), thus introducing a mutation that prevents caspase-3 cleavage. Consistent with our in vitro findings, primary neurons from WT mice showed SARM1 cleavage during apoptosis, whereas neurons from *Sarm1* D314A KI mice did not (Fig. 4*B*). This result supports our hypothesis regarding the importance of D314 in cleavage.

We next compared macrophages and T cells from *Sarm1* D314A KI mice to those from *Sarm1* KO mice. Both *Sarm1* KO and *Sarm1* D314A KI macrophages displayed reduced levels of apoptosis in response to the apoptotic trigger (Fig. 4*C*); similar results were observed in primary T cells (Fig. 4*D*). Importantly, the extent of reduction was similar in KI compared to KO macrophages, and in KI compared to KO T cells. These findings support the model that cleavage of SARM1 by caspase-3 activates SARM1 in those cells and contributes to apoptosis and that cleavage is required for SARM1’s action.

In primary sensory axons, the extent of Wallerian degeneration, where we did not observe SARM1 cleavage, was similar in neurons from WT and from *Sarm1* D314A mice (Fig. 4 *E* and *F*), underscoring the specificity of the cleavage-dependent apoptotic pathway and also reinforcing that uncleaved SARM1 can be activated by caspase-independent mechanisms.

As noted, in our cell culture paradigm axon degeneration from NGF-deprivation was not affected by *Sarm1* KO alone, so it was not surprising that it also was not affected by *Sarm1* D314A KI alone (Fig. 4 *G* and *H*). However, as also mentioned, *Sarm1* KO does provide protection against the degeneration seen in the presence of caspase inhibitor in our assay; in contrast, we found that *Sarm1* D314A KI does not provide this protection (Fig. 4 *G* and *H*). This reinforces that the axon degeneration-promoting action of SARM1 activated by NGF deprivation is partly independent of cleavage by caspase-3 at D314.

Collectively, our results have identified a role of SARM1 in promoting apoptotic cell death in T cells and macrophages. This role is executed by activation of SARM1’s NADase activity by caspase-3 cleavage. In neurons, SARM1 is also cleaved by caspase-3 during apoptotic axon degeneration, but cell death/axon degeneration promotion by SARM1 is not exclusively dependent on this pathway, implying that a distinct signaling pathway downstream of NGF deprivation can activate SARM1 in a cleavage-independent manner, in parallel to the apoptotic pathway (Fig. 4*I*).

## Discussion

While exploring mechanisms of SARM1-induced cell death, we found that SARM1 contributes to apoptosis downstream of activated caspase-3 in certain cells expressing SARM1, including Neuro-2a cells, as well as primary T cells and macrophages. We describe a mode of SARM1 activation through caspase-3-mediated cleavage at a specific site in the SARM1 autoinhibitory ARM domain. By engineering a mouse line in which SARM1 cleavage is blocked by mutation of that site, we show that this cleavage is required for SARM1 to contribute to macrophage and T cell apoptosis, whereas in neurons deprived of trophic support, which does activate a caspase-dependent pathway and cause SARM1 cleavage, SARM1 is also activated in parallel in a cleavage-independent manner.

### SARM1 Activation by Caspase Cleavage.

Several studies have sought to elucidate the physiological mechanisms by which SARM1 is activated, primarily focusing on the sensing by full-length SARM1 of NMN or NAD+ levels (22), as well as the NMN/NAD+ ratio(24). Sensing is proposed to be mediated by binding in the ARM domain (24), which also functions as the autoinhibitory domain of SARM1. A role for ADP-ribose conjugates and formation of superhelical filaments in SARM1 activation was also recently described (25–27), as was evidence that SARM1 can sense double-stranded DNA directly with its TIR domain, leading to activation (32).

In our study, we identified a physiological activation mode for SARM1 mediated by caspase-3 cleavage at D314, which removes most of the inhibitory ARM domain, including most of the NMN and NAD+ binding sites that might function as sensor sites. We hypothesize that this cleavage removes the intramolecular inhibition within the SARM1 protein, allowing oligomer formation and assembly into an activated state, and obviating the need for activation by NMN/NAD+ elevation. Further structural studies on this C-terminal fragment generated by cleavage will help assess this possibility, including whether it assembles into superhelical filaments.

Whether there are additional physiological mechanisms of SARM1 activation beyond those mentioned remains to be determined.

### What Is the Purpose of Activating SARM1 during Apoptosis?

Not all cells express physiological levels of SARM1, and only in cells that do could SARM1 contribute to apoptosis. For example, while T cells and macrophages express SARM1, which contributes to apoptosis in those cells, we found that HeLa cells do not normally express SARM1, but that adding it exogenously enhances cell death in those cells, indicating the capacity of these cells to use SARM1 to augment apoptosis.

This raises the question: In cells where SARM1 does contribute to apoptotic death upon caspase cleavage, is it there simply to help accelerate the death, or does it perform a specific role in the death process in those cells that is not needed in other cells that do not express SARM1 at significant levels?

Apoptosis has long been considered an immunologically silent form of cell death, eliciting minimal immune response when it occurs within tissues. This is largely due to the removal or processing of molecules that could trigger strong immune reactions. For instance, genomic DNA, known to robustly activate the innate immune response, is fragmented by the DFF40 endonuclease, thus eliminating its immunostimulatory effects (33, 34). Under normal conditions, DFF40 is kept inactive by its molecular chaperone, DFF45. However, during apoptosis, caspase-3/7 cleaves DFF45, releasing DFF40 and activating its endonuclease activity, causing fragmentation of genomic DNA (35).

This scenario suggests a possible role for the caspase-dependent activation of SARM1 reported here. Release of NAD+ from cells during inflammation has previously been associated with death of primary T cells in vivo (36). This raises the possibility that the pathway we have identified could serve to rapidly deplete intracellular NAD+, mitigating or abolishing the release of this “danger molecule” during apoptosis, and thereby reducing adverse effects and inflammation that might otherwise be associated with apoptotic cell death. Such a protective effect might be more important for some cells, such as T cells, than for other cells that are not as sensitive to extracellular NAD+. This in turn would provide a rationale for why only a subset of cells expresses physiological levels of SARM1 that contributes to apoptosis.

Further studies will help assess whether this proposed role in mitigating deleterious cell–cell communication is correct, or whether SARM1 activation during apoptosis plays some other role(s).

### Contributions of Caspase- and SARM1-Induced Degeneration in Developmental Axonal Degeneration.

Many studies of SARM1 have focused on primary neurons, where SARM1 is the key driver of injury-induced (Wallerian) axon degeneration, but its precise role in axon degeneration following trophic deprivation has been less clear, as different results have been seen in different studies. In some, deletion of *Sarm1* did not markedly affect the degeneration (4), but in others a contribution of SARM1 either was shown directly using *Sarm1* KO neurons (5) or was implied, as expression of the NAD+ producing fusion protein Wld^S^ or supplement with high-level NAD+ in the neuronal cultures, both known to inhibit SARM1’s action, were partially protective (37). A potential explanation for these differing results is that the studies used different culture conditions, including age of neurons used, how long they were cultured, whether neurons were dissociated or in tissue explants, and so forth—factors known to affect the time-course of degeneration and which could alter the relative contributions of different prodegenerative mechanisms. In addition, acute pharmacological caspase inhibition was found not to be as protective as constitutive *caspase-3* KO (30), further complicating comparison of studies.

Under the culture conditions used here (young neurons cultured for a short period in explants, whose axons undergo swift degeneration following trophic deprivation), we observed little protection from either acute pan-caspase inhibition or *Sarm1* KO alone, but strikingly strong protection when both manipulations were performed together. Protection in the presence of caspase inhibition was, however, not seen when using neurons from *Sarm1* D314A KI mice.

Two conclusions flow from this. First, while activation of caspases by trophic deprivation does cause SARM1 cleavage and presumably activation in these neurons, in our culture conditions SARM1-independent mechanisms downstream of caspases are sufficiently strong to mask any contribution SARM1 is making. Second, SARM1 is also activated in a cleavage-independent manner by trophic deprivation and can on its own drive the degeneration seen when caspase activation is blocked pharmacologically (Fig. 4*I*).

### Implications for Adult Neuropathy and Therapeutic Development in CIPN.

Our study has focused on embryonic neurons, but SARM1 was initially identified for its key role in adult neurons in injury-induced (Wallerian) degeneration, which is caspase-independent and, as our study shows, mediated by active uncleaved SARM1. In adults, SARM1 has also been extensively implicated as a major contributor to various neuropathies and neurodegenerative conditions, including chemotherapeutic-induced peripheral neuropathy (CIPN), a dose-limiting side effect of many cancer chemotherapeutics (38).

There has been considerable interest in understanding the molecular basis of CIPN, to help devise therapies to mitigate it. A model is provided by the degeneration of axons in response to chemotherapeutic drugs in cell culture and in mice, which has revealed that, while the underlying molecular mechanisms differ for different compounds, several converge on SARM1. For instance, the alkaloid vincristine, which inhibits microtubule formation, primarily triggers SARM1-dependent axon degeneration via a direct effect on axons, whereas bortezomib, a proteasome inhibitor, acts principally through the cell body to activate an apoptotic pathway that converges downstream on SARM1 in axons (39). Paclitaxel, which prevents microtubule breakdown, appears to activate axon degeneration via an IP_3_R1/calpain-dependent pathway that operates independently of apoptosis (40), but, again, converges on SARM1 as an effector (12).

These results have made SARM1 an attractive target for therapeutic development for CIPN, and both orthosteric and allosteric inhibitors are currently being evaluated for activity (41–43). Our results raise important considerations when choosing between these two classes of inhibitors. Allosteric inhibitors that target and stabilize the autoinhibitory ARM domain are expected only to block active uncleaved SARM1, not active cleaved SARM1, so they may be best to consider for chemotherapeutics that produce neuropathy in a caspase-independent manner, such as vincristine. Orthosteric inhibitors that target the NADase active site are expected to block both cleaved and uncleaved active forms of SARM1, as may any allosteric inhibitors that act on the cleaved form, so may be useful in all situations, but they will also interfere with caspase-activated SARM1 in other cells such as T cells and macrophages, with potential immune side effects.

The *Sarm1* D314A KI mice generated in this study provide a model to differentiate the contributions of caspase-dependent versus caspase-independent SARM1 activation and may help inform the development of potential therapies for CIPN and other forms of neural degeneration.

### SARM1 and Other Forms of Programmed Cell Death.

SARM1 has previously been reported to play a role in some cases of necroptosis (44), parthanatos (45, 46), and pyroptosis (29, 47). In the first two, signaling events appeared to converge on the increase of the NMN/NAD+ ratio and activation of SARM1 NADase activity, in line with the published SARM1 mode of action (44, 46). In pyroptosis, SARM1 was reported to interact with NLR family pyrin domain containing 3 (NLRP3) to inhibit inflammasome formation, caspase-1 activation, Gasdermin D (GSDMD) cleavage, and secretion of inflammatory cytokines but, somewhat paradoxically, to promote rather than inhibit pyroptotic cell death (29); whether NADase activity contributed was not reported, but in a different system SARM1 was reported to modulate cytokine production in both NADase-dependent and -independent manners (48). SARM1 was also reported to interact with another NOD-like receptor protein, NLRX1 [known to promote apoptosis in cancer cells (49)], to regulate Poly(ADP-ribose) polymerase 1 (PARP1) and caspase-3 cleavage in mouse embryonic fibroblasts undergo apoptosis (50). Again, the requirement of the SARM1 NADase activity in this case is yet to be determined. Of note, SARM1 was also found to function as an adaptor protein in Toll-like receptor (TLR) signaling pathways (51, 52), which could contribute to some of these instances of cell death (53). Future studies will help determine whether caspase-dependent SARM1 cleavage is required for its contribution to cell death in any of these systems.

## Methods

### Mice and Primary Cell Cultures.

Mice were bred and used according to IACUC protocols at Stanford University. WT mice (C57BL/6 and CD-1) were purchased from Charles Rivers. *Sarm1* KO mice were a gift from A. Ding (Weill Cornell Medicine, New York). *Sarm1* D314A KI mice were generated in house. For dorsal root ganglion (DRG) cultures, DRGs were dissected from E12.5 or E13.5 embryos harvested from pregnant dams and cultured in Neurobasal media with 2% B27 (Life Technologies), 2 mM glutamine, 100 U/mL penicillin, 0.45% (v/v) Glucose, 100 µg/mL streptomycin, 100 ng/mL NGF (Promega), and supplemented with mitotic inhibitors (5 µM 5-fluorouracil and 5 µM uridine, both from Sigma). DRGs were cultured in 24-well plates coated with poly-D-lysine (100 µg/mL Sigma) and laminin (10 µg/mL Gibco). Primary bone marrow derived macrophages (BMDM) were prepared from age and sex matched mice of different genotypes (WT, *Sarm1* KO or *Sarm1* D314 KI) following standard procedures (54). Briefly, bone marrow cells were harvested from mouse femur and tibia and differentiated in RPMI 1640 medium with 10% FBS, 100 U/mL penicillin, 100 µg/mL streptomycin, 2 mM L-glutamine and recombinant mouse M-CSF (10 ng/mL, PeproTech) for 7 d (with 10 mL fresh medium added per 10 cm dish on the fourth day of culture). Primary T cells were isolated using Pan T Cell Isolation Kit II, mouse (Miltenyi Biotec) from dissociated mouse spleens using Spleen Dissociation Kit, mouse (Miltenyi Biotech) following the manufacturer’s instructions. Primary T cells were cultured in RPMI 1640 medium with 10% FBS, 100 U/mL penicillin, 100 µg/mL streptomycin, and 2 mM L-glutamine. All cultures were maintained in standard cell culture incubators (Thermo Fisher Scientific) with 5% CO_2_ at 37 °C.

### Plasmids, Antibodies, and Reagents.

Mouse *Sarm1* complementary DNA (cDNA) was purchased from ORIGENE. Truncation mutants of SARM1 were generated using a standard PCR cloning strategy and inserted into the pCS2 vector with 3XFlag tag at the N terminus. Point mutations were generated using the Q5 Site-Directed Mutagenesis Kit (New England Biolabs). For stable expression of SARM1, the cDNA was cloned into the lentiviral vector FUIGW.

SARM1 antibody (clone 10G2) was a kind gift from Y.-P. Hsueh (Academia Sinica, Taipei, Taiwan) (55). Deletion analysis indicated that this antibody recognizes an epitope in the amino-terminal 27 amino acids of SARM1 (*SI Appendix*, Fig. S4). Monoclonal anti-Tubulin antibody was purchased from Sigma. Anti-NGF antibody was a kind gift from Genentech. Recombinant TNF was purchased from PeproTech. Cycloheximide was from Sigma. ABT-737 and the caspase inhibitor zVAD-FMK were purchased from Selleck Chemicals.

To identify potential SARM1 activators in Neuro-2a cells, the effect of different compounds on WT and KO Neuro-2a cells was assessed after 16 h in culture. A clear difference between effects on WT and KO Neuro-2a cells was seen with a combination of TNF (20 ng/mL) plus CHX (10 µg/mL). No obvious difference was seen with the following compounds: vincristine (Sigma, 1 µM), paraquat (Sigma, 333 µM), erastin (Sigma, 20 µM), anti-NGF antibody (25 µg/m), doxorubicin (20 µM), and paclitaxel (Sigma, 0.5 µM).

Protein samples were prepared by lysing the cells or DRG cultures using 1× SDS buffer (50 mM Tris-HCl, 100 mM DTT, 2% SDS, 1.5 mM bromophenol blue and 8% glycerol) and boiled for 30 min. Western blot was done by first separating protein using protein electrophoresis in 4 to 20% Mini-PROTEAN TGX Stain-Free Precast Gels (Bio-Rad). After visualizing the total protein signal with stainfree imaging (Bio-Rad Gel Doc XR+), the protein was then transferred from the gel into a PVDF membrane using Trans-Blot Turbo Transfer System (Bio-Rad). The membrane was blocked with 5% nonfat dry milk in Tris-Buffered Saline with Tween 20 (TBST, Bio-Rad) and then incubated with primary antibody for 1 h at room temperature and secondary antibody for 1 h diluted in TBST (5× TBST washes were performed after blocking, after primary, and after secondary antibody incubation). Protein signal is detected using Bio-Rad Gel Doc XR+ after incubating the membrane with Western Lightning Pro Chemiluminescent Substrate (PerkinElmer).

### Cell Culture and Transfection.

Neuro-2a, HEK293T cell, and HeLa cell were all purchased from ATCC and cultured according to the provided instructions. Transient transfection of HEK293T cells was performed using the jetPrime (Polyplus) reagent following the manufacturer’s instruction. For stable expression in HeLa cells, lentiviral plasmids (in FUIGW vector) with SARM1 or EGFP together with packaging plasmids pSPAX2 and pMD2.G were transfected into HEK293T cells. The supernatants (containing lentiviral particles) were collected 48 h after transfection and used to infect HeLa cells. GFP-positive cells were sorted out by flow cytometry. Protein transfections were performed using Neon Transfection System (Thermo Fisher Scientific). The electroporation condition for HEK293T cells was 1,100 V, 20 ms with 2 pulses. For experiments in Fig. 3*C*, HEK293T cells were transfected with plasmids expressing EGFP, WT SARM1, or SARM1 with the PreScission protease site (SARM1 PP) for 24 h. Cells were harvested and electroporated with Bovine Serum Albumin (BSA, 0.2 µg) as a control or with PreScission protease (1 unit) per 1 million cells, and cell survival was measured 20 h after electroporation.

### Measuring Cellular ATP, Caspase Activity, and SARM1 NADase.

Cellular ATP was measured by using CellTiter-Glo Luminescent Cell Viability Assay (Promega). Cellular caspase enzyme activity was measured using the Caspase-Glo Assay System (Promega). SARM1 NADase activity was measured by incubating recombinant SARM1 with 100 µM Nicotinamide 1, N6-ethenoadenine dinucleotide (ε-NAD) (56) for 1 h at 37 °C then measuring emission at 410 nm with excitation at 300 nm. Fluorescence gain was used to indicate the increase of SARM1 NADase.

### Generation of Neuro-2a *Sarm1* KO Cell Lines.

Cas9 (pLentiCas9-blast) and gRNA (pLentiGuide-Puro) expression plasmids were purchased from Addgene. gRNA targeting mouse *Sarm1* (GCGGCCGGAGGCCTCGACG) was cloned into the gRNA expression vector. To generate single cell derived *Sarm1* KO clones, 3 µg Cas9 expression plasmid, 1 µg gRNA expression plasmid and 1 µg EGFP expression plasmid (PDS127) were cotransfected into 6 million Neuro-2a cells. After 72 h, GFP-positive single cells were sorted into 96 cell plates using flow cytometry. Single clones were screened using a T7 endonuclease 1 (T7E1) assay and verified by SARM1 Western blot.

### In Vitro SARM1 Cleavage by Recombinant Caspases.

SARM1 was purified using anti-Flag M2 magnetic beads (Sigma) from HEK293T cells transfected with plasmid encoding 3XFlag-tagged SARM1. Recombinant SARM1 was kept on beads (~1 µg protein) and incubated with 1 unit recombinant active caspases (caspase-1, 2, 3, 7, 8, or 9, purchased from Enzo Life Sciences) in caspase assay buffer (Enzo Life Sciences) at 37 °C for 2 h. Cleavage was determined by protein electrophoresis of the incubated samples on Mini-PROTEAN TGX Stain-Free Precast Gels (Bio-Rad) and Coomassie Staining. The Edman degradation used to determine the caspase-3 cleavage site was performed by LakePharma.

### Generation of a *Sarm1* D314A KI Mouse.

The *Sarm1* D314A KI mouse was generated by the Stanford Transgenic, Knockout, and Tumor Model Center. Briefly, in vitro-translated Cas9 mRNA and gRNA together with a single chain recombination template (in which same-sense mutations were introduced to stop further Cas9 editing after successful recombination) were microinjected into C57BL/6 zygotes. KI founders were screened using T7E1 and verified by DNA sequencing. The gRNA sequence used was CCGCTTCGCCCGCTGCCTGG.

## Supplementary Material

Appendix 01 (PDF)

## Data Availability

Study data are included in the article and/or *SI Appendix*.
