# Supplementary material for: Caspase-3 cleaves and activates the NADase SARM1 to promote apoptosis, linking two cell death mechanisms
Source: Proc Natl Acad Sci U S A. 2026 Jan 23;123(4):e2528118123. doi: 10.1073/pnas.2528118123 (PMC12846840; doi:10.1073/pnas.2528118123)
Supplement: Supplementary file 1 — Appendix 01 (PDF) [file pnas.2528118123.sapp.pdf]

PNAS

Supporting Information for

Caspase-3 cleaves and activates the NADase SARM1 to promote apoptosis, linking two cell death mechanisms

Jianjin Shi[1], Ye Eun Kim[2], Nicolás José DeRuiter[3], Priyanka Kadav[4], Marc Tessier-Lavigne[5]

1. Department of Biology, Stanford University, Stanford, CA 94305, USA
jjshi@stanford.edu
2. Department of Biology, Stanford University, Stanford, CA 94305, USA
Current address: Mokdong apartment 1025-503, 400, Mokdongseo-ro, Yangcheon-gu, Seoul, Republic of Korea 08090
yeeun49@gmail.com
3. Department of Biology, Stanford University, Stanford, CA 94305, USA
Current address: Laboratory of Molecular Biophysics, The Rockefeller University, New York, NY, USA 10065; Laboratory of Molecular Pathogenesis, The Rockefeller University, New York, NY, USA 10065; Weill Cornell–Rockefeller–Sloan Kettering Tri-Institutional MD-PhD Program, New York, NY, USA
nderuiter@rockefeller.edu
4. Department of Biology, Stanford University, Stanford, CA 94305, USA
prikadav@stanford.edu
5. Department of Biology, Stanford University, Stanford, CA 94305, USA
tessier3@stanford.edu

Marc Tessier-Lavigne
Email: tessier3@stanford.edu

This PDF file includes:

Figures S1 to S4

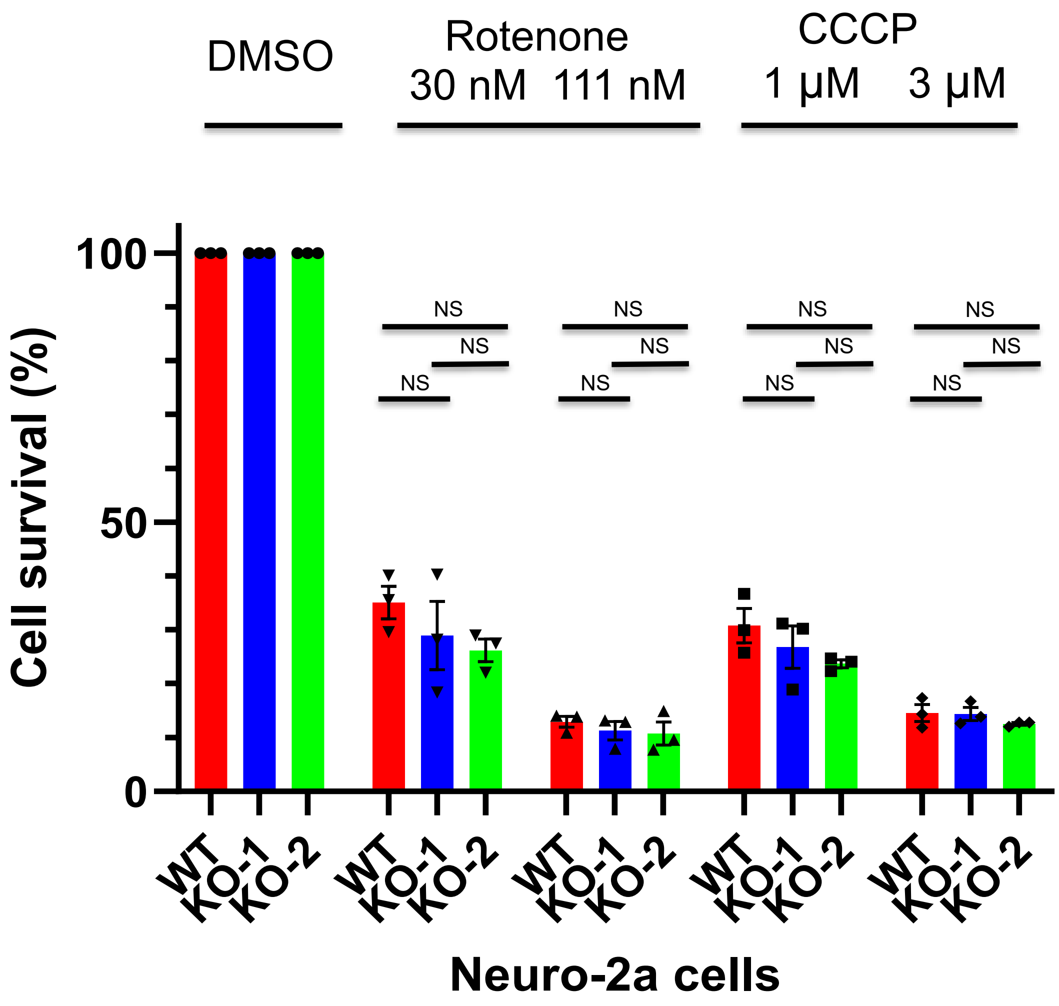

Fig. S1, Rotenone and CCCP trigger similar amount of cell death in WT and *Sarm1* KO Neuro-2a cells

WT Neuro-2a cells and two single-cell derived *Sarm1* KO lines (KO-1 and KO-2) were treated with DMSO, Rotenone or CCCP at indicated concentration for 22 hrs. Percent cell survival was determined by measuring total ATP level and normalizing to control (DMSO) treatment. Data are from three biological replicates. Student's t-test was used to determine statistical significance. NS, non-significant. Error bars represent SEM.

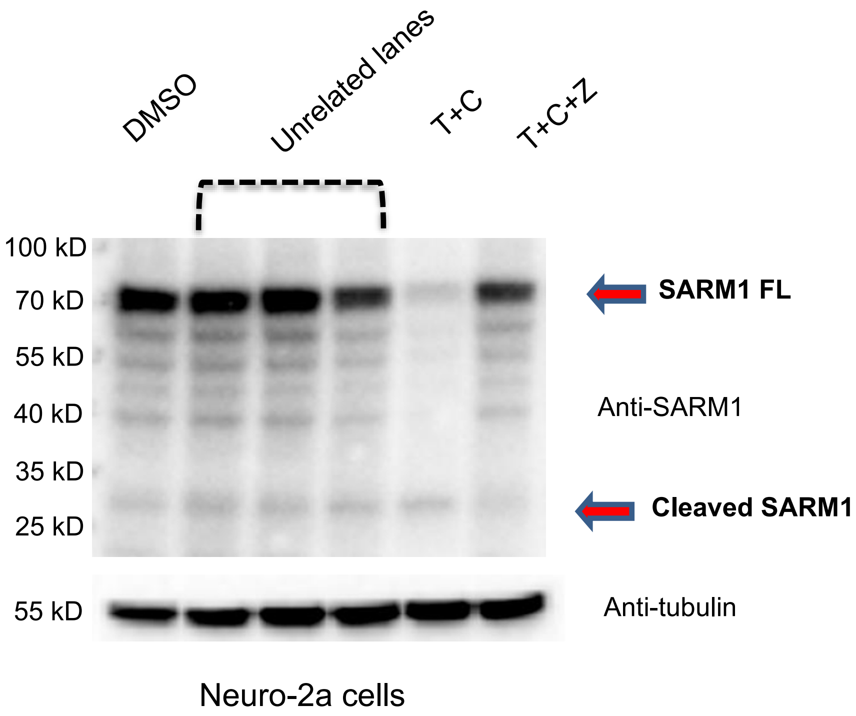

Fig. S2, Full Western blot image for Fig. 2D
Uncropped image for Fig. 2D. Unrelated lanes were cropped out in Fig. 2D.

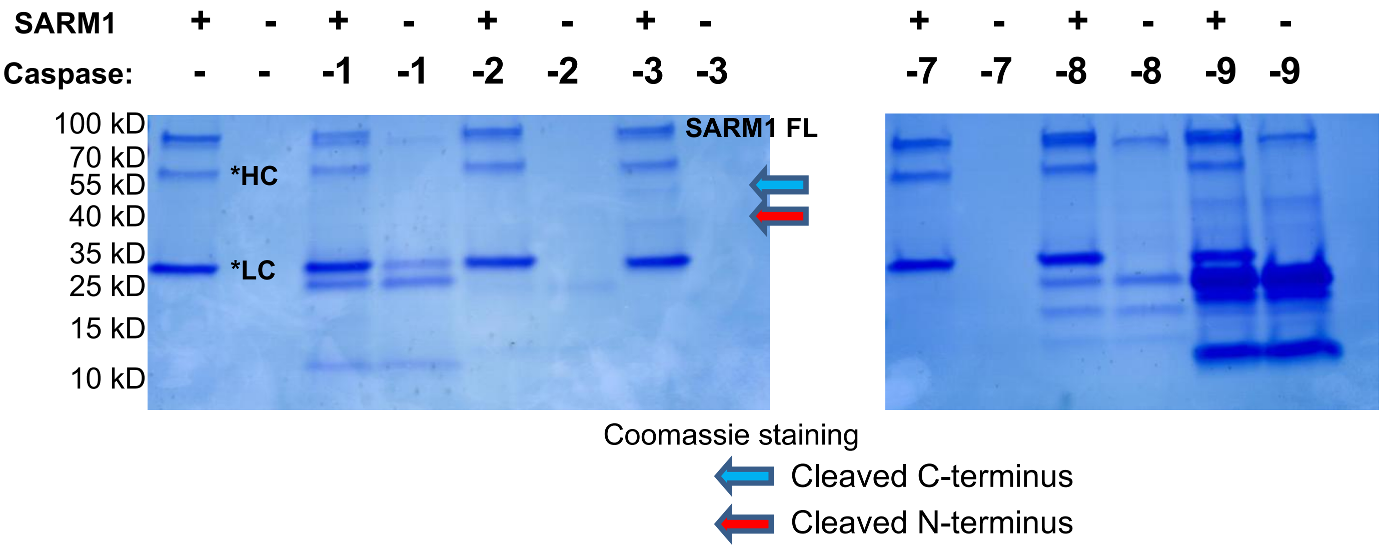

Fig. S3, *In vitro* cleavage of recombinant SARM1 by active caspases
Purified SARM1 (on beads) was incubated with active caspase-1, 2, 3, 7, 8, or 9. Cleavage was determined by protein electrophoresis and Coomassie staining. *HC, anti-Flag antibody heavy chain conjugated on beads, *LC: anti-Flag antibody light chain conjugated on beads. Data are representative of three independent experiments.

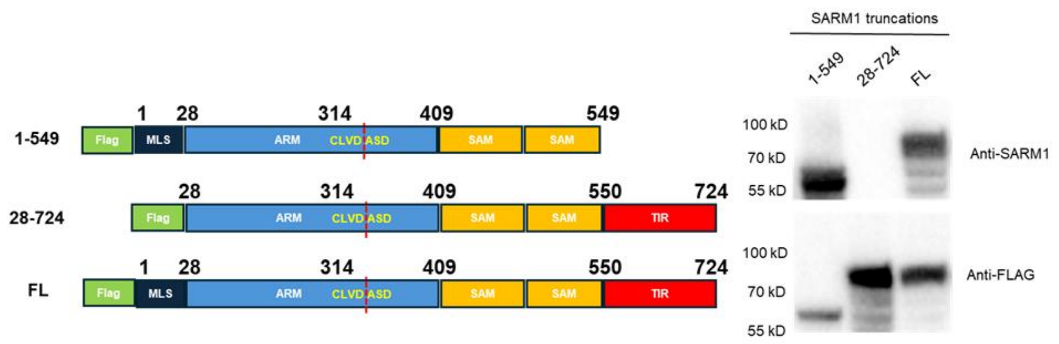

Fig. S4 Mapping the epitope of SARM1 antibody

HEK293T cells were transfected with indicated N terminal FLAG-tagged SARM1 truncations (shown in the diagram on the left) for 24 hours. Cell lysates were blotted with anti-SARM1 and anti-FLAG antibody (shown on the right). Note that the SARM1 antibody does not recognize the SARM1 truncation with the deletion of the 27 N-terminal amino acids (28-724). Data are representative of three independent experiments. MLS, mitochondrial localization signal. ARM, armadillo repeats. SAM, sterile alpha motif. TIR, Toll, Interleukin 1 receptor, and Resistance protein domain.
